# Statins dose-dependently exert a significant chemopreventive effect on colon cancer in patients with chronic obstructive pulmonary disease: A population-based cohort study

**DOI:** 10.18632/oncotarget.11263

**Published:** 2016-08-12

**Authors:** Ju-Chi Liu, Wen-Rui Hao, Yi-Ping Hsu, Li-Chin Sung, Pai-Feng Kao, Chao-Feng Lin, Alexander T.H. Wu, Kevin Sheng-Po Yuan, Szu-Yuan Wu

**Affiliations:** ^1^ Division of Cardiovascular Medicine, Department of Internal Medicine, Shuang Ho Hospital, Taipei Medical University, New Taipei, Taiwan; ^2^ Institute of Toxicology, College of Medicine, National Taiwan University, Taipei, Taiwan; ^3^ Department of Radiation Oncology, Wan Fang Hospital, Taipei Medical University, Taipei, Taiwan; ^4^ Department of Internal Medicine, School of Medicine, College of Medicine, Taipei Medical University, Taipei, Taiwan; ^5^ Department of Biotechnology, Hungkuang University, Taichung, Taiwan; ^6^ Ph.D. Program for Translational Medicine, Taipei Medical University, Taipei, Taiwan; ^7^ Department of Otorhinolaryngology, Wan Fang Hospital, Taipei Medical University, Taipei, Taiwan

**Keywords:** statins, chronic obstructive pulmonary disease, colon cancer

## Abstract

**Purpose:**

We evaluated the chemopreventive effect of statins on colon cancer in patients with chronic obstructive pulmonary disease (COPD) and identified the statin exerting the strongest chemopreventive effect.

**Methods:**

Using the National Health Insurance Research Database, we identified patients who received a COPD diagnosis in Taiwan between January 1, 2001, and December 31, 2012, and included them in the study cohort. Each patient was followed to assess the colon cancer risk and protective factors. A propensity score was derived using a logistic regression model to estimate the effect of statins by accounting for covariates predicted during the intervention (statins). To examine the dose–response relationship, we categorized statin doses into four groups in each cohort [<28, 28–90, 91–365, and >365 cumulative defined daily dose].

**Results:**

Compared with the statin nonusers, the adjusted hazard ratio (aHR) for colon cancer decreased in the statin users (aHR = 0.52, 95% confidence interval = 0.44, 0.62). Hydrophilic statins exerted a stronger preventive effect against colon cancer. Regarding the statin type, lovastatin, pravastatin, and fluvastatin nonsignificantly reduced the colon cancer risk in the patients with COPD. Compared with the statin nonusers, the aHRs for colon cancer decreased in the individual statin users (rosuvastatin, simvastatin, and atorvastatin: aHRs = 0.28, 0.64, and 0.65, respectively). In the sensitivity analysis, statins dose-dependently reduced the colon cancer risk.

**Conclusions:**

Statins dose-dependently exert significant chemopreventive effects on colon cancer in patients with COPD, with rosuvastatin exerting the largest chemopreventive effect.

## INTRODUCTION

Numerous epidemiological studies have indicated that tobacco smoking is the most crucial risk factor for chronic obstructive pulmonary disease (COPD) [1–6]. A considerably high prevalence of smoking has been reported among patients with COPD (approximately 54%–77% in patients with mild COPD and 38%–51% in patients with severe COPD) [7–10]. A 25-year follow-up study based on general population data from the Danish Death Register reported that 92% of patients who died from COPD had a regular smoking habit at the beginning of follow up, regardless of the timing of smoking cessation [2].

Smoking is associated with increased colon cancer incidence and mortality. A meta-analysis of 106 observational studies reported that the risk of colon cancer was higher among cigarette smokers than among those who never smoked [relative risk =1.18, 95% confidence interval (CI) = 1.11, 1.25] [11]. In addition, cigarette smoking is a risk factor for essentially all colonic polyps. Regarding adenomatous polyps, the risk is particularly high for advanced adenomas (i.e., large adenomas and those with dysplastic features) [12]. Moreover, smoking is a major risk factor for serrated polyps of the colon, including those that are hyperplastic and adenomatous [13–16]. In addition, smoking may increase the risk of colon cancer in patients with Lynch syndrome (i.e., hereditary nonpolyposis colorectal cancer) [17]. Therefore, most COPD patients with smoking habits may have a relatively increased risk of colon cancer compared with the general population.

Our previous observational study reported that statin use may reduce the overall risk of cancer and those of specific cancers [18]. The possible mechanisms underlying the decreased risk of cancer with statin use include the inhibition of downstream products of the mevalonate pathway [19–22], activation of tumor-specific apoptosis [23], and inhibition of the proteasome pathway [24]. Observational studies have suggested that statins have a protective effect against several cancers including colon cancer; however, the overall data are conflicting [25–29]. A modest reduction in the incidence of colon cancer, as a secondary endpoint, was observed in two large-scale clinical trials evaluating the benefits of pravastatin and simvastatin in coronary artery disease [30, 31].

The present study is the first to evaluate the incidence of colon cancer in patients with COPD and identify the statin type that most favorably reduces colon cancer risk in these patients.

## RESULTS

The COPD cohort comprised 43,802 patients: 10,086 (23%) used statins, whereas 33,716 (77%) did not (Table 1). The total follow-up duration was 194,933.6 and 80,239.4 person-years for statin users and nonusers, respectively (Table 2). The prevalence of preexisting medical comorbidities, namely diabetes (*P* < 0.001), hypertension (*P* < 0.001), and dyslipidemia (*P* < 0.001), and CCI (*P* < 0.001) was higher in the statin users than in the statin nonusers. In addition, significant differences were observed in the distribution of age; monthly income; urbanization level; and nonstatin lipid-lowering drug, aspirin, ACEI, and metformin use between statin and statin nonusers (Table 1). A higher proportion of statin nonusers used nonstatin lipid-lowering drugs, metformin, ACEI, and aspirin at ≥28 cDDDs, whereas most statin users used these drugs at <28 cDDDs. A higher proportion of statin users had a monthly income of NT$21,000–NT$33,300 or ≥NT$33,301 and resided in urban areas. Moreover, a larger proportion of statin users were women and elderly patients. Table 2 presents the colon cancer risk among the statin users and nonusers. After adjustment for age, sex, CCI, diabetes, hypertension, dyslipidemia, urbanization level, and monthly income in the PS analysis, we analyzed the colon cancer risk. Compared with the statin nonusers, the adjusted HRs (aHRs) of colon cancer decreased in the statin users (aHRs = 0.52, 95% CI = 0.44, 0.62). The stratified analysis revealed that the aHRs remained significantly decreased in the statin users, particularly in those aged 40–64 and ≥75 years, regardless of sex. Compared with the statin nonusers, the aHRs of colon cancer decreased in the statin users aged 40–64, 65–74, and ≥75 years (aHRs = 0.47, 0.54, and 0.48, respectively). After sex stratification, the aHR of colon cancer was lower in the statin users than in the statin nonusers (women: aHR = 0.55, 95% CI = 0.42, 0.72; men: aHR = 0.49, 95% CI = 0.39, 0.63). The effect of the reduced colon cancer risk was more predominant in the male statin users with COPD.

**Table 1:** Characteristic of the sample population

	Whole cohort (*n*=43802)		Patients with statin use (≥28 cDDD ; n=10086)		Patients without statin use (<28 cDDD ; n=33716)		*P*^a^
	n	%	n	%	n	%	
Age, years (Mean ± SD)	62.92 (13.18)		61.55 (10.97)		63.33 (13.74)		<0.001
40-54	14458	33.01	3180	31.53	11278	33.45	<0.001
55-64	9644	22.02	2899	28.74	6745	20.01	
65-74	10455	23.87	2777	27.53	7678	22.77	
≥75	9245	21.11	1230	12.20	8015	23.77	
Gender							
Female	19715	45.01	5150	51.06	14565	43.20	<0.001
Male	24087	54.99	4936	48.94	19151	56.80	
CCI Index^+^							
0	11279	25.75	2586	25.64	8693	25.78	<0.001
1	12597	28.76	3014	29.88	9583	28.42	
2	9075	20.72	2195	21.76	6880	20.41	
≥3	10851	24.77	2291	22.71	8560	25.39	
Diabetes							
No	33491	76.46	6819	67.61	26672	79.11	<0.001
Yes	10311	23.54	3267	32.39	7044	20.89	
Hypertension							
No	22067	50.38	4158	41.23	17909	53.12	<0.001
Yes	21735	49.62	5928	58.77	15807	46.88	
Dyslipidemia							
No	31731	72.44	5785	57.36	25946	76.95	<0.001
Yes	12071	27.56	4301	42.64	7770	23.05	
Nonstatin lipid-lowering drugs							
<28 cDDDs	39267	89.65	7212	71.51	32055	95.07	<0.001
28-365 cDDDs	3186	7.27	1923	19.07	1263	3.75	
>365 cDDDs	1349	3.08	951	9.43	398	1.18	
Metformin							
<28 cDDDs	35961	82.10	6286	62.32	29675	88.01	<0.001
28-365 cDDDs	2684	6.13	964	9.56	1720	5.10	
>365 cDDDs	5157	11.77	2836	28.12	2321	6.88	
ACEI							
<28 cDDDs	23928	54.63	3066	30.40	20862	61.88	<0.001
28-365 cDDDs	7925	18.09	1928	19.12	5997	17.79	
>365 cDDDs	11949	27.28	5092	50.49	6857	20.34	
Aspirin							
<28 cDDDs	28319	64.65	4161	41.26	24158	71.65	<0.001
28-365 cDDDs	7385	16.86	2296	22.76	5089	15.09	
>365 cDDDs	8098	18.49	3629	35.98	4469	13.25	
Level of Urbanization							
Urban	30539	69.72	7208	71.47	23331	69.20	<0.001
Suburban	8914	20.35	1920	19.04	6994	20.74	
Rural	4349	9.93	958	9.50	3391	10.06	
Monthly income (NT$)							
0	3464	7.91	795	7.88	2669	7.92	<0.001
1-21000	15001	34.25	3067	30.41	11934	35.40	
21000-33300	12904	29.46	3165	31.38	9739	28.89	
≥33301	12433	28.38	3059	30.33	9374	27.80	

a Comparison between statins use and statin nonusers

+ CCI Index: Charlson Comorbidity Index

**Table 2:** Risk of colon cancer among statin and non-statin in study cohort

All Group (n=43802	Patients without statin use (Total follow-up 194933.6 person-years)			Patients with statin use (Total follow-up 80239.4 person-years)			Adjusted HR^†^ (95% C.I.)
	No. of patients with cancer	Incidence rate (per 10^5^ person-years) (95% C.I.)		No. of patients with cancer	Incidence rate (per 10^5^ person-years) (95% C.I.)		
**Whole cohort**							
Colon Cancer	783	401.7	(373.5, 429.8)	163	203.1	(172.0, 234.3)	0.52(0.44, 0.62)***
**Age, 40-64**^a^							
Colon Cancer	304	261.6	(232.2, 291.0)	73	144.6	(111.4, 177.8)	0.47(0.36, 0.62)***
**Age, 65-74**^b^							
Colon Cancer	261	585.0	(514.1, 656.0)	66	303.1	(229.9, 376.2)	0.54(0.41, 0.72)***
**Age, ≥75**^c^							
Colon Cancer	218	639.4	(554.6, 724.3)	24	300.6	(180.3, 420.8)	0.48(0.31, 0.74)***
**Female**^d^							
Colon Cancer	277	317.0	(279.6, 354.3)	78	186.5	(145.1, 227.9)	0.55(0.42, 0.72)***
**Male**^e^							
Colon Cancer	506	470.5	(429.5, 511.5)	85	221.3	(174.2, 268.3)	0.49(0.39, 0.63)***

a Total follow-up 116228.5 person-year for patients without statin use and 50476.0 for patients with statin use.

b Total follow-up 44612.9 person-year for patients without statin use and 21778.3 for patients with statin use.

c Total follow-up 34092.2 person-year for patients without statin use and 7985.1 for patients with statin use.

d Total follow-up 87389.9 person-year for patients without statin use and 41828.7 for patients with statin use.

e Total follow-up 107543.7 person-year for patients without statin use and 38410.7 for patients with statin use.

C.I.: confidence interval

HR: hazard ratio

† Main model is adjusted for age, sex, Charlson comorbidity index, diabetes, hypertension, dyslipidemia, level of urbanization, Monthly income in propensity score.

Statins dose-dependently reduced the colon cancer risk in the different cDDD subgroups, and the main model was adjusted for age, sex, CCI, diabetes, hypertension, dyslipidemia, urbanization level, and monthly income in the PS analysis (Table 3). Lipophilic statins included simvastatin, lovastatin, atorvastatin, and fluvastatin. Hydrophilic statins included pravastatin and rosuvastatin. Both lipophilic and hydrophilic statins reduced the colon cancer risk in the patients with COPD in a dose-dependent manner (*P* for trend < 0.001; Table 3). When the patients with COPD had 91–365 or >365 cDDDs of statins, the hydrophilic statins exerted a larger preventive effect on the reduction in colon cancer risk. Regarding individual statin users, lovastatin, pravastatin, and fluvastatin nonsignificantly reduced the colon cancer risk. Compared with the statin nonusers, the aHRs of colon cancer decreased in individual statin users (rosuvastatin, simvastatin, and atorvastatin: aHRs = 0.28, 0.64, and 0.65, respectively). Our results revealed that different statin types had different efficacies in reducing colon cancer in the patients with COPD.

**Table 3:** Incidence rate and adjusted HRs of colon cancer associated with statin use during the follow-up period in COPD patients

Variable	No. of patients	No. of person-years	No. of patients with colon cancer	Incidence rate (per 10^5^ person-years) (95% C.I.)		Adjusted HR (95%C.I.)	P for trend
Total statin use							
Nonuser (<28 cDDDs)	33716	194933.6	783	401.7	(373.5, 429.8)	1.00	<0.001
User (≥28 cDDDs)	10086	80239.4	163	203.1	(172.0, 234.3)	0.52(0.44, 0.62)***	
28-90 cDDDs	2346	17095.6	49	286.6	(206.4, 366.9)	0.72(0.54, 0.96)*	
91-365 cDDDs	3215	24193.1	59	243.9	(181.6, 306.1)	0.62(0.47, 0.80)***	
>365 cDDDs	4525	38950.7	55	141.2	(103.9, 178.5)	0.36(0.27, 0.47)***	
Lipophilia statin use^†^							
Nonuser (<28 cDDDs)	35008	204288.0	799	391.1	(364.0, 418.2)	1.00	<0.001
User (≥28 cDDDs)	8794	70885.0	147	207.4	(173.9, 240.9)	0.64(0.53, 0.77)***	
28-90 cDDDs	2296	17069.8	42	246.0	(171.6, 320.5)	0.69(0.50, 0.94)*	
91-365 cDDDs	3012	23258.7	59	253.7	(188.9, 318.4)	0.77(0.58, 1.01)	
>365 cDDDs	3486	30556.4	46	150.5	(107.0, 194.0)	0.50(0.36, 0.67)***	
Hydrophilia statin use^†^							
Nonuser (>28 cDDDs)	39878	242812.7	902	371.5	(347.2, 395.7)	1.00	<0.001
User (≥28 cDDDs)	3924	32360.4	44	136.0	(95.8, 176.1)	0.48(0.35, 0.66)***	
28-90 cDDDs	1122	8876.1	19	214.1	(117.8, 310.3)	0.72(0.45, 1.13)	
91-365 cDDDs	1531	12432.2	14	112.6	(53.6, 171.6)	0.41(0.24, 0.69)***	
>365 cDDDs	1271	11052.0	11	99.5	(40.7, 158.3)	0.36(0.20, 0.66)***	
Individual statin use (≥28 cDDDs)^‡^							
Simvastatin	3418	28625.0	48	167.7	(120.2, 215.1)	0.64(0.47, 0.87)**	
Lovastatin	2109	18281.5	47	257.1	(183.6, 330.6)	1.02(0.75, 1.38)	
Atorvastatin	5484	44678.1	84	188.0	(147.8, 228.2)	0.65(0.52, 0.83)***	
Fluvastatin	1510	12855.7	29	225.6	(143.5, 307.7)	0.93(0.63, 1.35)	
Pravastatin	1501	12654.5	27	213.4	(132.9, 293.8)	0.89(0.60, 1.32)	
Rosuvastatin	2741	22641.7	18	79.5	(42.8, 116.2)	0.28(0.17, 0.45)***	

Main model is adjusted for age, sex, Charlson comorbidity index, diabetes, hypertension, dyslipidemia, level of urbanization, Monthly income in propensity score.

† Lipophilia statins include simvastatin, lovastatin, atorvastatin, and fluvastatin. Hydrophilia statins include pravastatin and rosuvastatin.

‡ The HRs of individual statin users (≥ 28 cDDDs) were compared with Total statin of nonusers (<28 cDDDs).

In the sensitivity analysis, adjustments were made to estimate the association of age; sex; CCI; diabetes; hypertension; dyslipidemia; urbanization level; monthly income; and nonstatin lipid-lowering drug, metformin, ACEI, and aspirin use with the incidence of colon cancer in different models. Table 4 shows that the effects of statins remained significant in the subgroups of various covariates in the main model adjusted for PSs. Statins dose-dependently reduced the colon cancer risk in all subgroups and in the main model with additional covariates (nonstatin lipid-lowering drugs, metformin, ACEI, and aspirin use). All aHRs indicated that statins significantly and dose-dependently reduced the colon cancer risk in all subgroups, regardless of age, sex, comorbidity, or drug use (*P* < 0.001). Thus, our data revealed that statins exert a chemopreventive effect against colon cancer dose-dependently.

**Table 4:** Sensitivity analysis of adjusted HRs of statin in risk reduction of colon cancer

	Statin use				P for trend
	<28 cDDDs	28-90 cDDDs	91-365 cDDDs	>365 cDDDs	
	Adjusted HR (95%C.I.)	Adjusted HR (95%C.I.)	Adjusted HR (95%C.I.)	Adjusted HR (95%C.I.)	
**Main model^†^**	1.00	0.72(0.54, 0.96)^*^	0.62(0.47, 0.80)^***^	0.36(0.27, 0.47)^***^	<0.001
**Additional covariates**^‡^					
Main model ^+^ Nonstatin	1.00	0.73(0.54, 0.97)^*^	0.63(0.48, 0.82)^***^	0.37(0.28, 0.49)^***^	<0.001
Main model ^+^ Metformin	1.00	0.72(0.54, 0.96)^*^	0.63(0.48, 0.82)^***^	0.39(0.29, 0.51)^***^	<0.001
Main model ^+^ ACEI	1.00	0.73(0.55, 0.98)^*^	0.66(0.50, 0.86)^**^	0.42(0.31, 0.56)^***^	<0.001
Main model ^+^ Aspirin	1.00	0.72(0.54, 0.97)^*^	0.64(0.49, 0.84)^**^	0.39(0.29, 0.52)^***^	<0.001
**Subgroup effects**					
Age, years					
40-64	1.00	0.59(0.37, 0.95)^*^	0.58(0.38, 0.87)^**^	0.36(0.25, 0.54)^***^	<0.001
65-74	1.00	0.86(0.55, 1.35)	0.64(0.42, 0.98)^*^	0.35(0.22, 0.55)^***^	<0.001
≥75	1.00	0.80(0.42, 1.50)	0.54(0.28, 1.06)	0.24(0.10, 0.58)^**^	<0.001
Sex					
Female	1.00	0.73(0.47, 1.14)	0.67(0.46, 1.00)	0.40(0.27, 0.59)^***^	<0.001
Male	1.00	0.73(0.50, 1.07)	0.57(0.40, 0.83)^**^	0.32(0.22, 0.48)^***^	<0.001
CCI Index^+^					
0	1.00	0.46(0.23, 0.93)^*^	0.46(0.26, 0.83)^*^	0.35(0.21, 0.59)^***^	<0.001
1	1.00	0.74(0.44, 1.24)	0.57(0.34, 0.94)^*^	0.45(0.28, 0.71)^***^	<0.001
2	1.00	0.70(0.39, 1.27)	0.57(0.32, 1.01)	0.36(0.20, 0.63)^***^	<0.001
≥3	1.00	0.99(0.57, 1.71)	0.83(0.51, 1.36)	0.16(0.06, 0.39)^***^	<0.001
Diabetes					
No	1.00	0.67(0.48, 0.95)^*^	0.59(0.43, 0.82)^**^	0.34(0.23, 0.48)^***^	<0.001
Yes	1.00	0.84(0.49, 1.43)	0.62(0.39, 0.98)^*^	0.36(0.23, 0.56)^***^	<0.001
Dyslipidemia					
No	1.00	0.68(0.47, 0.98)^*^	0.52(0.36, 0.75)^***^	0.35(0.24, 0.51)^***^	<0.001
Yes	1.00	0.77(0.48, 1.26)	0.73(0.49, 1.09)	0.35(0.23, 0.54)^***^	<0.001
Hypertension					
No	1.00	0.86(0.57, 1.29)	0.44(0.26, 0.73)^**^	0.38(0.24, 0.62)^***^	<0.001
Yes	1.00	0.62(0.41, 0.93)^*^	0.69(0.50, 0.94)^*^	0.33(0.23, 0.46)^***^	<0.001
NonStatin					
<28 cDDDs	1.00	0.77(0.56, 1.05)	0.63(0.46, 0.86)^**^	0.34(0.24, 0.48)^***^	<0.001
28-365 cDDDs	1.00	0.48(0.19, 1.22)	0.69(0.37, 1.29)	0.47(0.26, 0.83)^*^	0.013
>365 cDDDs	1.00	1.17(0.30, 4.54)	0.47(0.12, 1.83)	0.31(0.10, 0.99)^*^	0.028
Metformin					
<28 cDDDs	1.00	0.68(0.48, 0.95)^*^	0.63(0.46, 0.87)^**^	0.31(0.21, 0.47)^***^	<0.001
28-365 cDDDs	1.00	0.86(0.40, 1.85)	0.55(0.24, 1.22)	0.34(0.13, 0.86)^*^	0.010
>365 cDDDs	1.00	1.07(0.45, 2.55)	0.90(0.47, 1.75)	0.68(0.40, 1.15)	0.150
ACEI					
<28 cDDDs	1.00	0.75(0.49, 1.15)	0.56(0.35, 0.90)^*^	0.27(0.13, 0.53)^***^	<0.001
28-365 cDDDs	1.00	0.91(0.54, 1.53)	0.67(0.38, 1.18)	0.72(0.42, 1.24)	0.102
>365 cDDDs	1.00	0.77(0.42, 1.43)	1.01(0.67, 1.54)	0.51(0.34, 0.76)^***^	0.003
Aspirin					
<28 cDDDs	1.00	0.77(0.52, 1.14)	0.59(0.38, 0.89)^*^	0.45(0.29, 0.69)^***^	<0.001
28-365 cDDDs	1.00	0.75(0.44, 1.27)	0.69(0.41, 1.17)	0.52(0.30, 0.92)^*^	0.010
>365 cDDDs	1.00	0.79(0.38, 1.64)	0.90(0.55, 1.46)	0.38(0.23, 0.63)^***^	<0.001

* *p*<0.05

** *p*<0.01

*** *p*<0.001

HR: hazard ratio

+ CCI Index: Charlson Comorbidity Index

† Main model is adjusted for age, sex, Charlson comorbidity index, diabetes, hypertension, dyslipidemia, level of urbanization, Monthly income in propensity score.

‡ The models were adjusted for covariates in the main model as well as each additional listed covariate.

## DISCUSSION

COPD is characterized by both airway and systemic inflammation [36]. Epidemiological studies have reported that an increase in the levels of systemic inflammatory markers, mainly C-reactive protein (CRP), interleukin 6 (IL-6), and fibrinogen, predicts poor outcomes in COPD, higher propensity to infective exacerbations, and higher mortality [37–39]. COPD significantly enhances this inflammatory disposition by acting as a recurring proinflammatory stimulus to the pulmonary and immune systems. An increase in systemic inflammation in patients with COPD has been linked to progressive loss of lung function [40–46] and several cancer types [46–48]. The strongest association of chronic inflammation with malignant diseases is in colon carcinogenesis arising in individuals with inflammatory bowel disease [49]. Mucosal inflammation may be an independent risk factor for colon cancer [50]. The connection between inflammation and tumorigenesis is well-established and has been reported in genetic, pharmacological, and epidemiological studies in the last decade [51–54]. Moreover, inflammation is likely involved in other forms of sporadic and heritable colon cancer [52]. Furthermore, >90% of patients with COPD are habitual smokers [2, 55], and smoking is associated with an increased incidence of colon cancer [11]. Smoking is also a risk factor for essentially all colonic polyp types [12, 13]. Therefore, patients with severe COPD may have a high colon cancer risk. Furthermore, inclusion of patients without COPD may reveal the different colon cancer preventive effects of statins [56]. The Global Initiative for Chronic Obstructive Lung Disease guidelines recommend that a fixed combination of inhaled corticosteroids or long-acting b2 agonists should be considered for patients with group C or D COPD, who are highly symptomatic or have a high risk of complications [57]. Here, the presence of a baseline pneumonia event and frequent baseline and recent AE were used as surrogates to control for COPD severity. Because our study included only those COPD patients with more than two instances of AEs or any AE requiring hospitalization within the 12 months between enrollment and the index date, the enrolled patients likely represented those who essentially required inhaled corticosteroid therapy [57, 58]. The development of a safe and effective chemopreventive strategy is warranted for patients with severe COPD in order to reduce the risk of colon cancer.

The beneficial effects of statins are not completely understood. Studies have reported that statins reduce inflammation and baseline inflammatory marker levels, cause thrombogenicity, and reverse endothelial dysfunction [59–61]. Randomized studies have reported that statins reduce levels of inflammatory markers such as CRP and IL-6 [62, 63]. However, the mechanisms by which statins interfere in the inflammatory response remain unclear. A possible mechanism is the impairment of inflammatory cell adhesion through the inhibition of the beta-2 integrin leukocyte function antigen-1 [64, 65]. Other potentially contributing factors include decreased lipidation of intracellular proteins, expression of major histocompatibility complex class II molecules on antigen-presenting cells in response to interferons, and subsequent activation of T lymphocytes [65, 66]. A statin-induced decrease in CRP levels may be partly mediated by the decreased monocyte expression of proinflammatory cytokines that stimulate the release of acute phase proteins [67]. This may be the mechanism by which statins reduce the colon cancer risk in patients with COPD and high systemic inflammation [36].

Colon cancer is the most common malignancy in Taiwan [68]. Age is a major risk factor for sporadic colon cancer. Large bowel cancer is uncommon in people younger than 40 years; thus, we included only those patients who were aged ≥40 years and we observed a larger chemopreventive effect against colon cancer in patients with COPD aged 40–64 and ≥75 years (Table 2). This finding indicates that statins exert a chemopreventive effect in middle-aged and elderly patients with COPD. After sex stratification, the aHR was lower in the statin users than in the statin nonusers (women: aHR = 0.55, 95% CI = 0.42, 0.72; men: aHR = 0.49, 95% CI = 0.39, 0.63). Moreover, in our study, the effect of colon cancer risk reduction was predominant in the male statin users with COPD. This finding is consistent with that of another study that reported primary prevention of coronary heart disease through statin therapy in women; this risk reduction was weakly significant (HR = 0.89; 95% CI = 0.79, 1.00; *P* = 0.05) [69]. However, the reasons for the differences in the effects have not been clarified thus far.

In our study, statins dose-dependently reduced the colon cancer risk in different cDDD subgroups, and the main model was adjusted for age, sex, CCI, diabetes, hypertension, dyslipidemia, urbanization level, and monthly income in the PS analysis (Table 3). Both lipophilic and hydrophilic statins reduced the colon cancer risk in the patients with COPD in a dose-dependent manner (*P* for trend < 0.001; Table 3). In those who used statins at a 91–365 or >365 cDDDs, the hydrophilic statins exerted a larger preventive effect in the reduction of the colon cancer risk. Regarding the various statin types, lovastatin, pravastatin, and fluvastatin nonsignificantly reduced the colon cancer risk in the patients with COPD. Compared with the statin nonusers, the aHRs of colon cancer decreased in the individual statin users (aHRs = 0.28, 0.64, and 0.65 for rosuvastatin, simvastatin, and atorvastatin, respectively). Therefore, our results indicate that different statin types have different efficacies in reducing colon cancer risk in patients with COPD. The efficacy of statins in reducing this risk is compatible with the lipid-lowering potency of different statins. Rosuvastatin, atorvastatin, and simvastatin cause the highest percentage change in low-density lipoprotein cholesterol [70–72] and have the largest chemopreventive effect against colon cancer in patients with COPD. The efficacy of individual statins in reducing inflammation and baseline inflammatory marker levels, causing thrombogenicity, and reversing endothelial dysfunction are also distinct [73–75]. This is the first study to evaluate which statin type has the largest chemopreventive effect in reducing colon cancer risk in patients with COPD. Our study results can facilitate the appropriate selection of more favorable statins in future clinical studies.

*In vitro* and *in vivo* data suggest that angiotensin II is involved in promoting cancer development and that ACEI use and reduced colon cancer cell growth are associated [76, 77]. Metformin use significantly reduces the incidence of pancreatic, liver, breast, and colon cancers [78]. Regular use of aspirin and other nonsteroidal anti-inflammatory drugs decreases the risk of adenomatous polyps and colorectal cancer [79, 80]. Nonstatin lipid-lowering drugs and statins differ regarding their mechanisms of action and lipid-lowering degrees and types. Therefore, we used a sensitivity analysis to include possible unmeasured confounding factors to distinguish the independent anticolon cancer effects of statins. In the present study, a higher proportion of statin nonusers used nonstatin lipid-lowering drugs, metformin, ACEI, and aspirin at ≥28 cDDDs, whereas most statin users used these drugs at <28 cDDDs. Furthermore, moderate-to-high (28–365 cDDDs or >365 cDDD) use of aspirin, metformin, and ACEI masked the chemopreventive effect of statins in reducing the colon cancer risk (Table 4). In our sensitivity analysis, the aHRs of statins in reducing the colon cancer risk of patients with COPD were significant when the statin dose was increased to >365 cDDDs. These outcomes may explain the independent chemopreventive effects of aspirin, metformin, ACEI, nonstatin lipid-lowering drugs, and statins [81, 82]. This is the first study to report that statins exert a dose-dependent chemopreventive effect against colon cancer in patients with COPD.

Our study has some potential limitations. We could not eliminate the bias of additional risk factors related to COPD and colon cancer such as personal or family history of sporadic colon cancer, obesity, alcohol use, excessive processed meat consumption, physical activity, and smoking status. Therefore, a large-scale randomized trial with a suitable regimen in selected patients should compare standard approaches to obtain this crucial information. However, methodological concerns may obscure the precise association between these factors and colon cancer risk. In addition, a low socioeconomic status is associated with an increased risk of colon cancer [83]. In our study, we used the PS analysis to match age, sex, CCI, diabetes, hypertension, dyslipidemia, urbanization level, and monthly income between groups. Urbanization level and monthly income are nonvalidated alternatives to lifestyle and environmental factors. Moreover, the diagnoses of colon cancer and all other comorbid conditions depended completely on the ICD-9-CM codes. Nevertheless, the National Health Insurance Administration randomly reviews medical records and interviews patients to validate diagnoses. The hospitals with outlier diagnoses and practices may be audited and subsequently heavily penalized if malpractice or discrepancies are discovered. Another limitation is that information on several unmeasured confounders, such as body mass index, smoking, alcohol intake, and other over-the-counter drug use (associated with colon cancer), is unavailable in the NHIRD. However, considering the magnitude and significance of the observed effects, these limitations have unlikely compromised the final results. Finally, our study is not a prospective randomized blinded study; hence, a cause–effect relationship could not be established. The findings of this study suggest that statins dose-dependently exert a significant chemopreventive effect against colon cancer in patients with COPD. Additional randomized studies are required to verify these findings.

## PATIENTS AND METHODS

The National Health Insurance program, implemented in 1995, currently provides comprehensive health insurance coverage to >98% of the 23 million residents of Taiwan. We used data from the National Health Insurance Research Database (NHIRD), in which the distributions of age, sex, and health care costs does not differ significantly from those in the general population. Data in the NHIRD that could identify patients or care providers, including medical institutions and physicians, are encrypted before being forwarded to the National Health Research Institutes for database construction and are further encrypted before being released to researchers. Theoretically, querying the data alone to identify people at any level by using this database is impractical [18].

**Figure 1:**
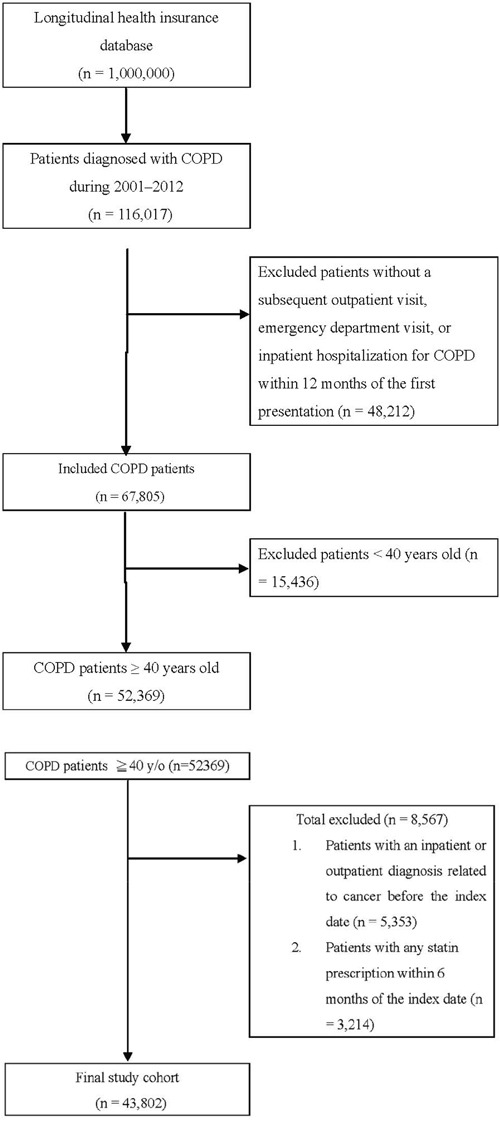
Patient selection flowchart

From the NHIRD, all patients who received a COPD diagnosis at health care facilities in Taiwan (*n* = 116,017) between January 1, 2001, and December 31, 2012 were identified according to International Classification of Diseases, Ninth Revision, Clinical Modification (ICD-9-CM) codes 491, 492, 496, and A-code A325. Among the patients who fulfilled the selection criteria of COPD, those with two episodes of acute exacerbation (AE) within 1 year or one episode of AE requiring hospitalization were identified, with the first AE date defined as the enrollment date. AE was defined as emergency department visits or admissions with ICD-9-CM codes 491, 492, 496, and A-code A325 plus prescription of systemic corticosteroids. We excluded all patients without a subsequent outpatient visit, emergency department visit, or inpatient hospitalization for COPD within 12 months of the first presentation (*n* = 48,212) because they were considered to not have COPD (Figure 1). The index date was noted as 365 days after the enrollment date. The baseline frequency of AE was calculated during the 1-year period from the enrollment to the index date [32]. In addition, we excluded 15,436 patients who were aged <40 years (*n* = 52,369), had a history of any inpatient or outpatient diagnosis related to cancer before the enrollment date (*n* = 5,353), or had any statin prescription within 6 months before the enrollment date (*n* = 3,214). Finally, we included 43,802 patients with COPD in the study cohort and followed them for an 11-year period. Of the 43,802 patients with COPD, 10,086 used statins and 33,716 did not.

Each patient was followed to assess the colon cancer risk and protective factors. We evaluated the demographic characteristics of age and sex; comorbidities of diabetes, hypertension, and dyslipidemia; Charlson comorbidity index (CCI); urbanization level; monthly income; and nonstatin lipid-lowering drug, metformin, aspirin, and angiotensin-converting enzyme inhibitor (ACEI) use (Supplementary Table S1). The date of COPD diagnosis was considered the index date of statin use. This study aimed to evaluate the preventive effects of statin use in patients with COPD and a high colon cancer risk. The primary endpoint was colon cancer risk, and the secondary endpoints were the benefits of different doses and types of statins used in patients with COPD. The defined daily dose (DDD), as defined by the World Health Organization, is a measure of the prescribed drug amount. DDD is the assumed average maintenance dose per day of a drug consumed for its main indication in adults [18]. Because the duration of the prescription refill card is 3 months, we categorized the DDD (entire duration of observation for each patient) of statins into four groups in each cohort [<28, 28–90, 91–365, and >365 cumulative DDDs (cDDDs)] to examine the dose–response relationship. Patients who received statins at <28 cDDDs were defined as statin nonusers (Tables 2–4) [33]. Furthermore, we categorized statins used into individual statin groups in each cohort to examine the preventive effect of different statin types (Table 3).

A propensity score (PS) was derived using a logistic regression model to estimate the effect of statins by accounting for covariates predicted during the intervention (statins). All potential confounders (Supplementary Table S1) were included in the list of regressors (C statistic: 0.678). This method is used in observational studies to reduce selection bias [34]. The covariates in the main model were adjusted for the PSs of age (40–54, 55–64, and ≥75 years), sex, CCI, diabetes, hypertension, dyslipidemia, urbanization level, and monthly income (0, NT$1–NT$21,000, NT$21,000–NT$33,300, and ≥NT$33,301; Table 2). The endpoint was the incidence of colon cancer (ICD-9-CM codes 153.0–153.9) among the statin and statin nonusers with a subsequent outpatient visit, emergency department visit, or inpatient hospitalization for colon cancer within 12 months; patients who did not receive statins served as the reference arm. The cumulative incidence of colon cancer in the patients with COPD with or without statin use was estimated using the Kaplan–Meier method.

A time-dependent Cox proportional hazard model was used to calculate the hazard ratios (HRs) of colon cancer among the statin users and nonusers. In the multivariate analysis, the HRs were adjusted for age, sex, CCI, diabetes, hypertension, dyslipidemia, urbanization level, and monthly income. A stratified analysis was conducted to evaluate the effect of statin use on age and sex (Table 2). All analyses were conducted using SAS software (version 9.3; SAS, Cary, NC, USA); two-tailed *P* < 0.05 was considered significant. In sensitivity analyses, external adjustments clarify the effects of drugs and other covariates in epidemiological database studies [35]. Hence, in the sensitivity analysis conducted in the present study, adjustments were made to estimate the association of age and sex; diabetes, dyslipidemia, hypertension, CCI, and anxiety disorder; and nonstatin lipid-lowering drug, metformin, aspirin, and ACEI use with the incidence of colon cancer in different models. The models stratified by the use of different drugs were adjusted for covariates in the main model and each additional covariate (Table 4).

## CONCLUSIONS

Statins dose-dependently exert a significant chemopreventive effect against colon cancer in patients with COPD, with rosuvastatin, atorvastatin, and simvastatin exerting the largest chemopreventive effects.

## SUPPLEMENTARY TABLE


