# Supplementary material for: Statins dose-dependently exert a significant chemopreventive effect on colon cancer in patients with chronic obstructive pulmonary disease: A population-based cohort study
Source: Oncotarget. 2016 Aug 12;7(40):65270–83. doi: 10.18632/oncotarget.11263 (PMC5323154; doi:10.18632/oncotarget.11263)
Supplement: Supplementary file 1 [file oncotarget-07-65270-s001.pdf]

Statins dose-dependently exert a significant chemopreventive effect on colon cancer in patients with chronic obstructive pulmonary disease: A population-based cohort study

SUPPLEMENTARY TABLE

Supplementary Table S1: Candidate variables for logistic regression model

Candidate variable	Odd ration	95% Confidence Interval	
≥75 y/o	1.294	1.216	1.377
65-74 y/o	1.051	0.981	1.125
55-64 y/o	0.449	0.412	0.490
female	0.766	0.730	0.803
CCI Index ≥3	0.893	0.839	0.951
CCI Index 2	0.796	0.743	0.854
CCI Index 1	0.577	0.536	0.620
Diabetes	1.485	1.405	1.570
Hypertension	1.699	1.612	1.790
Dyslipidemia	2.180	2.069	2.297
Urban	0.982	0.924	1.044
Suburban	0.987	0.907	1.074
income ≥33301	0.838	0.763	0.920
income21000-33300	0.956	0.867	1.056
income 1-21000	0.930	0.840	1.030

+CCI Index: Charlson Comorbidity Index
